# Screening of a chemogenetic library unveils dual-stage antiplasmodials with low cytotoxicity

**DOI:** 10.1128/aac.01613-25

**Published:** 2026-10-05

**Authors:** Laura Torres, Matthew E. Fishbaugher, Melanie Lam, Alexander T. Chao, Calla Martyn, Luying Pei, Debapriya Sengupta, Maxime Dauphinais, Jonathan E. Gable, Srinivasa P. S. Rao, Steffen Renner, W. Armand Guiguemde, Sebastian A. Mikolajczak, Anke Harupa, Gabriel Mitchell

**Affiliations:** 1 Open Innovation at Global Health Disease Area, Biomedical Research, Novartis 98558, Emeryville, California, USA; 2 Global Health Disease Area, Biomedical Research, Novartis 98558, Emeryville, California, USA; 3 Discovery Sciences, Biomedical Research, Novartis 98558, Emeryville, California, USA; 4 Global Discovery Chemistry, Biomedical Research, Novartis 98558, Emeryville, California, USA; 5 Discovery Sciences, Biomedical Research, Novartis, Basel, Switzerland; The Children's Hospital of Philadelphia, Philadelphia, Pennsylvania, USA

**Keywords:** *Plasmodium*, malaria, liver, blood, drug discovery, global health

## Abstract

The global effort to eradicate malaria requires the development of drugs with novel mechanisms of action and differentiated pharmacological profiles. Both liver and blood phases of *Plasmodium* infection are essential for disease progression and transmission, making them primary targets for pharmacological intervention. In this study, we established a dual-luciferase reporter assay to simultaneously assess *Plasmodium berghei* liver-stage development and host cell viability. Screening a library of bioactive small molecules with annotated human targets revealed several inhibitors with potent liver-stage activity and minimal cytotoxicity. Further profiling against *Plasmodium falciparum* asexual blood stages identified compounds with dual-stage activity, underscoring their potential utility for development as either prophylactic or therapeutic agents. Knowledge of their human targets will facilitate investigation into host- or parasite-directed antimalarial mechanisms, thereby accelerating both biological insight and rational drug development. Our findings constitute a valuable resource for research on malaria biology and the discovery of next-generation antiplasmodials.

## INTRODUCTION

Malaria remains a global health challenge, affecting hundreds of millions annually and causing life-threatening complications, particularly in young children, pregnant women, travelers, and individuals with HIV (1). Most malaria cases are attributed to infection with *Plasmodium falciparum* (*Pf*), predominantly found in sub-Saharan Africa (1). However, *Plasmodium vivax* also accounts for a significant portion of malaria cases, exhibits a more widespread distribution, and causes clinical relapses (1, 2). *Plasmodium* parasites have repeatedly demonstrated their adaptability to environmental challenges and, despite the deployment of resources for antimalarial discovery, novel therapeutics are still required to face the emergence of drug resistance and support the global effort to eradicate malaria (3).

The complex lifecycle of *Plasmodium* provides several opportunities for pharmacological intervention (3, 4). Transmission begins when *Plasmodium* sporozoites are injected into humans by *Anopheles* mosquitoes. Shortly after the bite, sporozoites infiltrate the liver, where they invade hepatocytes and establish a parasitophorous vacuole, a specialized compartment that supports intracellular development by leveraging both host and parasite biological processes (5). Within this vacuole, parasites differentiate and replicate extensively via schizogony (5). In addition to forming replicative liver-stage schizonts, certain species such as *P. vivax* can produce hypnozoites. Hypnozoites are quiescent forms that persist for weeks to months before activating, leading to clinical relapses and continued transmission (2). Upon completion of liver-stage development, thousands of merozoites are released into the bloodstream, where they invade red blood cells and initiate the asexual replication cycle responsible for the symptomatic phase of malaria. A subset of these asexual blood-stage parasites differentiates into gametocytes, which can be taken up by mosquitoes during a blood meal and complete sexual reproduction in the mosquito midgut (3, 6).

Although the liver phase is clinically silent, it is a critical bottleneck in the parasite lifecycle and an attractive target for chemoprevention. Liver-stage inhibitors can block the establishment of blood-stage infection, prevent symptom onset, and reduce transmission. Moreover, because parasite burden is low at this stage, liver-targeting compounds may carry a reduced risk of resistance development. Dual-stage inhibitors, which are active against both liver and asexual blood stages (BS), offer additional flexibility, as they may be developed either as preventive agents or as treatments, depending on clinical needs and resistance management strategies (3, 4).

Phenotypic screening of compound libraries against *Plasmodium* parasites has been instrumental in antimalarial drug discovery (4). However, the largest and most comprehensive screens have focused on asexual blood stages (7–9), potentially biasing hit identification toward compounds that target blood stage-specific pathways (10–12). These screens typically involve testing large numbers of structurally diverse compounds, with only a minor fraction selected for subsequent medicinal chemistry optimization to improve potency, selectivity, and pharmacological properties, ultimately yielding molecules with robust *in vivo* efficacy (4, 13). Elucidating the mechanism of action for hits from phenotypic screens can be challenging, especially for compounds with a high barrier to resistance (14). However, once validated, biological targets can guide more focused drug discovery, a process that can be further accelerated through computational and structure-based approaches (4, 13).

In this study, we developed a dual-luciferase assay to rapidly assess both cytotoxicity and inhibitory activity of compounds against the liver stages (LS) of *P. berghei* (*Pb*), a model organism that causes malaria in rodents and readily infects human hepatoma cells (5, 15). Using this assay, we screened a library of bioactive compounds, including molecules with known mechanisms of action in human cells, and identified several hits with activity against *P. berghei* liver stages and minimal cytotoxicity. Some hits also inhibited *P. falciparum* asexual blood stages, suggesting either direct antiparasitic activity or the targeting of host pathways common to both hepatocytes and red blood cells. Other hits were only active against *P. berghei* liver stages, potentially acting on liver stage-specific host or parasite pathways. Overall, this study provides detailed information on novel compounds with antiplasmodial activity, including chemical structures and known human targets. These compounds represent promising starting points for the development of next-generation antiplasmodials.

## MATERIALS AND METHODS

### Cell culture

The human hepatoma Huh7 cell line (Japanese Collection of Research Bioresources Cell Bank) was grown in Dulbecco’s modified Eagle’s medium (DMEM) with high glucose (Gibco) supplemented with 10% heat-inactivated fetal bovine serum (FBS, Corning), 100 U/mL penicillin-streptomycin (Gibco), and 1% GlutaMAX (Gibco) (hereafter referred to as Huh7 medium) at 37°C and 5% CO_2_.

### Generation of Renilla luciferase-expressing Huh7 cells

Stable Renilla luciferase-expressing Huh7 cells (Huh7_Rluc_) were generated with lentivirus from BPS Bioscience (79565-G). Briefly, cells were transduced with a multiplicity of infection of 2 in Huh7 medium containing 8 µg/mL of polybrene (AmericanBio), followed by centrifugation at 800 × *g* for 1 h at 37°C, with low acceleration and break. One day post-transduction, lentiviruses were removed, and fresh cell culture medium was added to cells. Transduced cells were then selected with 250 µg/mL of geneticin (G418) for 5 weeks.

### *P. berghei* liver-stage infection assay

One day prior to infection, WT Huh7 or Huh7_Rluc_ cells were seeded at a density of 8,000 cells per well in a 384-well white solid-bottom plate (Greiner, 781080), unless otherwise specified. *Anopheles stephensi* mosquitoes infected with luciferase-expressing *P. berghei* ANKA strain GFP-Luc_ama1-eef1a_ (*Pb*_Fluc_) (16) were produced by the SporoCore (University of Georgia, UGA), and sporozoites were isolated by microdissection of salivary glands, as previously described with modifications (17, 18). Unless otherwise specified, cells were infected with 2,000 sporozoites per well in Roswell Park Memorial Institute 1640 (RPMI, Gibco) medium containing 20% heat-inactivated FBS (Corning). Infection was facilitated by centrifugation at 330 × *g* for 3 min at room temperature (RT), using low acceleration and braking settings. Cells were incubated with sporozoites for 2 h at 37°C and 5% CO_2_. The culture medium was then replaced with Huh7 medium, and compounds were added prior to further incubation for 48 h under the same conditions.

### Dual-luciferase reporter assay

The Dual-Glo luciferase assay (Promega) was performed as specified by the provider with modifications. Briefly, medium from cell cultures was replaced with 20 µL of Dual-Glo luciferase reagent per well. Plates were incubated at RT for 10 min in the dark and shaken prior to measuring firefly luciferase (Fluc) luminescence signal. The Stop & Glo buffer was then mixed 1:1 with DMEM without phenol red (Gibco), and 40 µL was added per well. Plates were then incubated at RT in the dark for 20 min and shaken prior to measuring the Renilla luciferase (Rluc) luminescence signal. Luminescence signals were read using CLARIOstar (BMG Labtech) or PHERAstar FSX (BMG Labtech) plate readers.

### Rluc assay

The Renilla-Glo luciferase assay system (Promega) was used as specified by the provider with modifications. Briefly, Huh7_Rluc_ cells were seeded at different concentrations in a 384-well white solid-bottom plate (Greiner) and incubated for 1 day at 37°C and 5% CO_2_. The cell culture medium was then removed and replaced with 10 µL of Dulbecco’s phosphate-buffered saline (D-PBS, Gibco) and 10 µL of Renilla-Glo per well. Plates were shaken, incubated at RT in the dark for 10 min, and the luminescence signal was read using a CLARIOstar plate reader.

### ATP cell viability assay

Promega’s CellTiter-Glo 2.0 (CTG) assay kit was used as specified by the provider with modifications. Briefly, 8,000 Huh7_Rluc_ cells per well were seeded in a 384-well white solid-bottom plate (Greiner) and incubated for 1 day at 37°C and 5% CO_2_. Cells were then treated with compounds for 2 days: the cell culture medium was replaced with 20 µL of CellTiter-Glo reagent per well, plates were shaken and incubated at RT in the dark for 10 min, and the luminescence signal was read using a CLARIOstar plate reader.

### Screening of compounds against *P. berghei* using the dual-luciferase reporter assay

At 2 h post-sporozoite infection, cell culture medium was replaced, and samples were treated with a library of 6,044 compounds at a final concentration of 10 μM using the Echo 650 Acoustic Liquid Handler (Beckman). This library comprises bioactive compounds with known human gene targets, including some compounds from the previously published Novartis Moa Box collection (19). Some, but not all, of the information associated with this library is publicly accessible and can be retrieved using InChIKeys provided for 4,273 compounds (Data S1). Two days post-treatment, the dual-luciferase reporter assay was performed, and luminescence signals were measured. Robust Z′ (RZ′) values were calculated for each plate using luminescence signal values from DMSO-treated infected wells and DMSO-treated uninfected (Fluc) or empty (Rluc) wells. The data from plates with one or more RZ′ below 0.2 were excluded. Percent activities were determined by normalizing the values of each well to DMSO-treated infected wells (0% inhibition) and DMSO-treated uninfected or empty wells (100% inhibition for Fluc and Rluc, respectively) and averaged for two to four independent experiments. Experiments were initially performed using three consecutive shipments of infected mosquitoes. Because sporozoite yield varied between shipments and constituted the limiting factor for experimental throughput, each shipment was used to screen as many compound plates as possible. As a result, the number of independent experiments per compound varied. A fourth shipment was subsequently used to prioritize repeat testing of selected plates, ensuring that all compounds were tested in at least two independent experiments. For hit determination, the median values of DMSO-treated infected wells minus 4× (Fluc, threshold = −84.24%) and 3× (Rluc, threshold = −41.64%) the robust standard deviations were applied as thresholds. Out of the 198 hits, 126 publicly available compounds were further characterized and included in this publication.

### *P. falciparum* asexual blood-stage assay

*P. falciparum* 3D7 (BEI Resources, MRA-102) parasite cultures were grown with complete medium (RPMI 1640 medium [10.4 g/L] with 0.5% AlbuMAX II, 200 μM hypoxanthine, 50 mg/L gentamicin sulfate, 35 mM HEPES, 2.0 g/L sodium bicarbonate, and 11 mM glucose) and human erythrocytes at 37°C and 5% CO_2_, as previously described (14). The antiplasmodial activity of compounds was measured according to a modified SYBR Green cell proliferation assay, as previously described (14).

### Structural comparison of human ATR and *Plasmodium* proteins

A structural search was conducted to identify proteins similar to human ATR (UniProt ID Q13535; PDB ID 5YZ0) among all *P. falciparum* proteins using Foldseek (20) incorporated into UniProt (search parameters = AlphaFold/UniProt50v4 + PDB100 20240101, mode: 3Di/AA, taxonomic filter: *Plasmodium falciparum*, iterative search off). The top *P. falciparum* hit, an AlphaFold model for a phosphatidylinositol 4-kinase [PI(4)K, UniProt ID Q8I406], showed structural alignment between residues 1280 and 1556 and human ATR residues 2320 and 2632. The structural similarity between *Plasmodium* PI(4)K_1280–1556_ and human ATR_2320–2632_ was confirmed using PyMOL (root mean square deviation: 1.832 Å; The PyMOL Molecular Graphics System, Version 3.1.3, Schrödinger, LLC).

### PI(4)K enzymatic assay

A PI(4)K enzymatic assay was performed as previously described (21). Briefly, the assay was performed for 1 h at RT in a 384-well black plate using the Transcreener ADP_2_ FP detection kit (BellBrook) and a final assay volume of 10 µL containing 1 nM of purified *P. vivax* PI(4)K protein, 10 mM Tris, pH 7.5, 1 mM DTT, 10 µM ATP, 5 mM Mn^2+^, 0.2% Triton X-100, and 11 µM L-α-phosphatidylinositol (dissolved in 3% *n*-octylglucoside). The reaction was then stopped by adding 10 µL of detection mix containing 1× stop buffer, 4 nM AMP Alexa Fluor 633 tracer, and 20 µg/mL of ADP antibody. Fluorescence polarization measurements were performed on the Infinite M1000 plate reader (Tecan) with λ_ex_ = 635 nm and λ_em_= 680 nm (bandwidth, 20 nm).

### Compounds, dose-response treatments, and analyses

All compounds were obtained from the Novartis internal archive and resuspended in DMSO. For standard dose-response experiments, compounds were prepared as 7- or 10-point threefold or half-log serial dilution series and were transferred from master plates into assay plates using a mosquito HV liquid handler (SPT Labtech), with a maximum concentration of 10 μM. Results were normalized (100%, DMSO-treated samples) and plotted with the curve fit setting “log(inhibitor) vs normalized response” on GraphPad Prism (version 9.5) or with DAVID Helios (version 3.2.0.364) using a standard logistic regression model, as previously described (22). Unless otherwise stated in figure legends, curve fitting was performed using Helios. To enable ranking of hit compounds and integration of results from independent experiments, IC_50_ and CC_50_ values were transformed prior to calculation of medians, selectivity indexes (SIs), and IC_50_*Pf* BS / IC_50_*Pb* LS ratios (see Data S1 for detailed information). Briefly, data outside the limits of experimental detection were imputed to half the next lower or higher untested concentration, based on the serial dilution scheme used for each assay. Data were subsequently subjected to log10-transformation, and median values were calculated. To compute SIs and IC_50_*Pf* BS / IC_50_*Pb* LS ratios, values were reverted to their original scale. To ensure accuracy and avoid misleading interpretation, median values were re-expressed together with their corresponding experimental detection limits. As with compound screening, dose-response validation of hit compounds using the dual-luciferase reporter assay was conducted with four shipments of infected mosquitoes. The final shipment was used to repeat selected plates, ensuring that each compound was tested at least twice.

The reliability of the data presented in this study is limited by variations in the number of independent experiments performed for each compound and assay. The numbers of technical replicates and independent experiments are indicated in the figure legends or in Data S1.

### Annotation of hit compounds

Data for hit compounds were gathered from ChEMBL v35 (downloaded on 2024-12-01) and PubChem (downloaded on 2025-01-22). Target scores were determined as previously described (23).

### Preparation of graphs and artwork

BioRender (biorender.com), GraphPad Prism (v9.5), ChemDraw Professional (v23.1.1.3), and Adobe Illustrator (v29.8.1) were used to create figures.

## RESULTS

### A dual-luciferase reporter assay for assessing *P. berghei* liver-stage development and host cell viability

**Fig 1:**
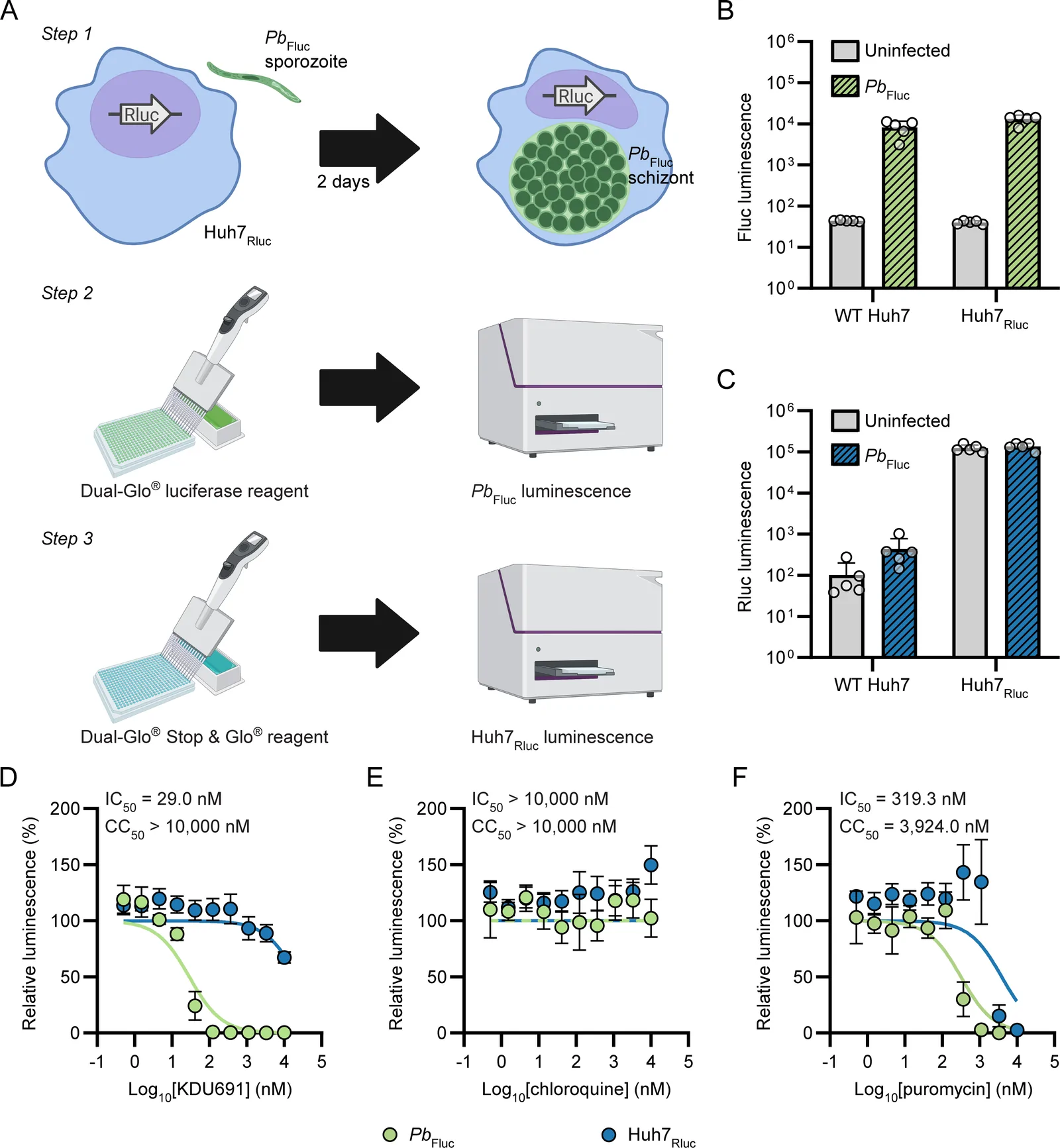
A dual-luciferase reporter assay for simultaneous assessment of *P. berghei* liver-stage development and host cell viability. (**A**) Schematic overview of the dual-luciferase reporter assay. (Step 1) Renilla luciferase (Rluc)-expressing Huh7 (Huh7_Rluc_) cells were infected with firefly luciferase (Fluc)-expressing *P. berghei* (*Pb*_Fluc_) and incubated for 2 days prior to measuring (Step 2) *Pb*_Fluc_ and (Step 3) Huh7_Rluc_ luminescence. Fluc (**B**) and Rluc (**C**) luminescence of WT Huh7 and Huh7_Rluc_ cells infected with *Pb*_Fluc_ for 2 days. Results are unnormalized and presented as means and SDs from one experiment with five technical replicates (*n* = 5). Dose-response effect of the liver-stage inhibitor KDU691 (**D**), the blood-stage inhibitor chloroquine (**E**), and the cytotoxic control puromycin (**F**) on Fluc and Rluc luminescence signals. Half-maximal inhibitory concentration (IC_50_) and half-maximal cytotoxic concentration (CC_50_) values were determined for each compound using GraphPad Prism, and data were pooled across experiments. See Data S1 for single and averaged IC50 and CC50 values from each independent experiment. Data points are normalized and expressed as means and SEMs from four independent experiments (*N* = 4, with 2–4 technical replicates). 100% corresponds to DMSO-treated infected samples.

A dual-luciferase assay was customized to simultaneously evaluate parasite growth and host cell viability during the liver phase of *Plasmodium* infection (Fig. 1A). As a reporter of host cell viability, the Renilla luciferase (Rluc) gene was introduced into Huh7 hepatoma cells via lentiviral transduction, yielding a luminescence signal that strongly correlated with cell number (Fig. S1A). These Rluc-expressing cells (Huh7_Rluc_) were then infected with *P. berghei* sporozoites expressing the firefly luciferase (Fluc) gene (*Pb*_Fluc_) (16) for a 2-day incubation period (Fig. 1A, step 1). Parasite and host signals were then quantified using the Dual-Glo Luciferase Assay System, which enables sequential measurement of Fluc (Fig. 1A, step 2) and Rluc (Fig. 1A, step 3) luminescence. The Dual-Glo assay is optimized for high-throughput analysis of Fluc and Rluc signals, providing increased signal stability but lower sensitivity compared with other reagents such as the Bright-Glo Luciferase Assay System (24–26), which is commonly used in *P. berghei* liver-stage screening experiments (27, 28). Despite its lower sensitivity, both Fluc and Rluc signals produced by the Dual-Glo assay were elevated by more than two orders of magnitude compared with uninfected or untransduced controls (Fig. 1B and C; Fig. S2). In the representative experiment shown in Fig. 1B and C, mean signal-to-noise ratios were 326-fold for Fluc (Fluc_infected_ / Fluc_uninfected_ in Huh7_Rluc_) and 313-fold for Rluc (Rluc_infected_ in Huh7_Rluc_ / Rluc_infected_ in WT Huh7). Moreover, the Fluc signal exhibited a strong positive correlation with the number of *Pb*_Fluc_ sporozoites used for infection (Fig. S1B), demonstrating the assay’s sensitivity to parasite load.

To confirm the suitability of this assay for compound screening during liver-stage infection, we first evaluated three reference compounds: KDU691 (Fig. 1D), a known inhibitor of liver stages (21); chloroquine (Fig. 1E), which is active against asexual blood stages but not liver stages (29); and puromycin (Fig. 1F), a cytotoxic control compound. As anticipated, KDU691 reduced the *Pb*_Fluc_ signal with IC_50_ values of 24.3–29.0 nM, consistent with previously reported values (21, 27), and showed negligible cytotoxicity toward Huh7_Rluc_ cells, with CC_50_ values typically exceeding 10 µM (Fig. 1D; Fig. S3D and Data S1). In line with expectations, chloroquine did not reduce either luminescence signal (Fig. 1E; Fig. S3F and Data S1) (27), whereas puromycin affected both parasite and host cell signals (Fig. 1F; Fig. S3G and Data S1) (see GNF-*Pf*-2016 in reference 30). It is noteworthy that puromycin inhibited *Pb*_Fluc_ at lower concentrations than those affecting host cell viability, with IC_50_ values of 231.0–319.3 nM, compared with CC_50_ values of 2,994–3,924 nM in Huh7_Rluc_ cells. This likely reflects the faster replication rate of liver-stage parasites relative to host cells. Potency against *Pb*_Fluc_ was also in agreement with previously reported data for other established antimalarials, including cabamiquine (27, 31), atovaquone (27, 32, 33), and KAF179 (11) (Fig. S3 and Data S1). KAE609 showed only weak activity against *P. berghei* liver stages (IC_50_ = 4,607 nM) (Fig. S3E and Data S1), consistent with earlier findings (11, 34) but contrasting with a recent report by Nguyen et al. (27). Notably, KAE609 showed minor cytotoxicity at higher concentrations, which may have contributed to the observed weak *Pb*_Fluc_ activity (Fig. S3). We further confirmed the cell viability data for these antimalarials using an orthogonal assay (CellTiter-Glo, CTG) that quantifies viable cells based on ATP levels (Fig. S3 and Data S1). Beyond the variation that may be expected when measuring cytotoxicity using different readouts (35–37), we found that results were consistent across both assays. Overall, these results demonstrate that the dual-luciferase assay is a reliable and sensitive platform for evaluating compound activity against *P. berghei* liver stages and host cells.

### Screening of a bioactive library against *P. berghei* liver stages using the dual-luciferase reporter assay

**Fig 2:**
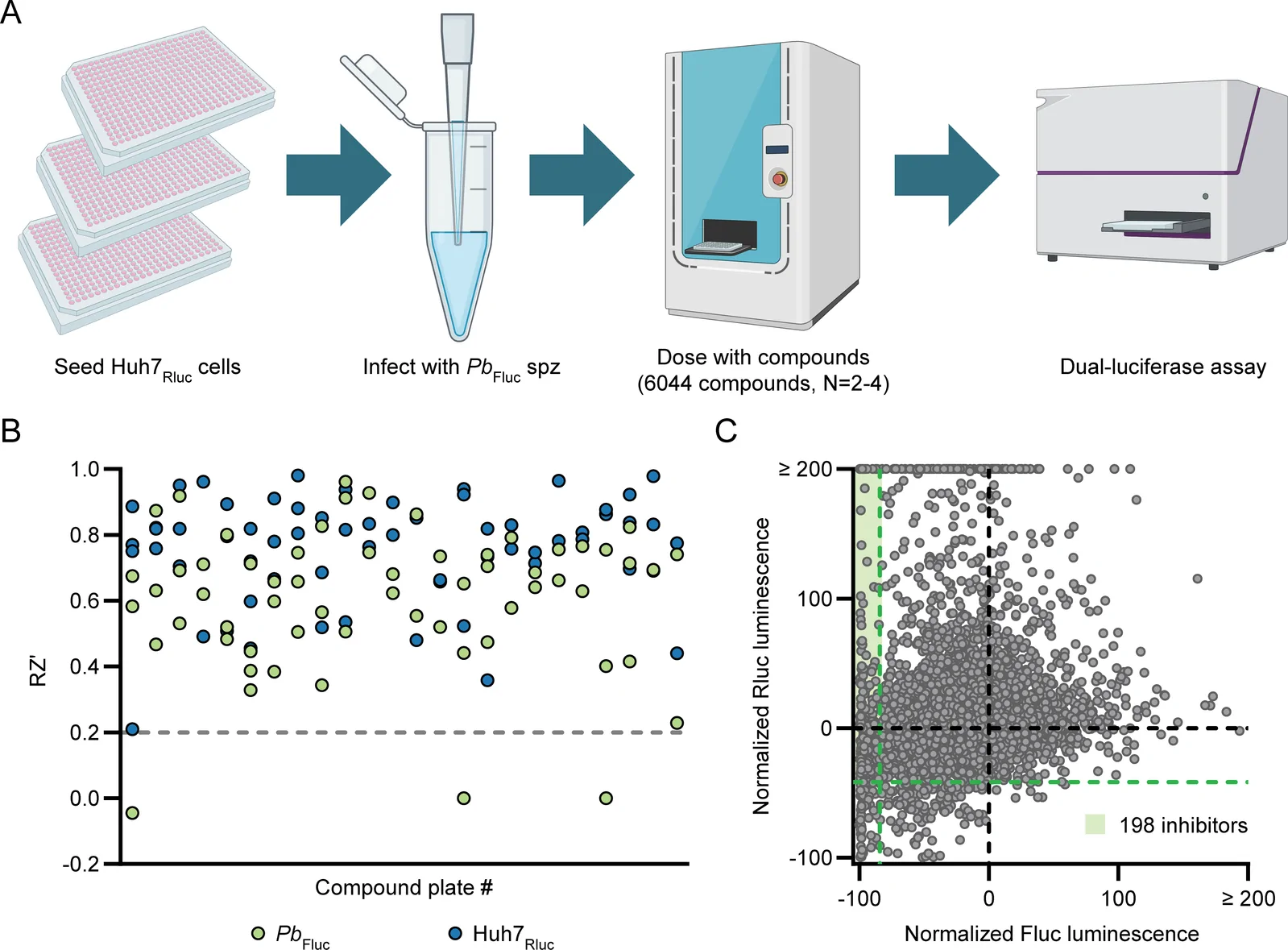
Screening of a chemogenetic library against *P. berghei* liver stages using the dual-luciferase reporter assay. (**A**) Diagram outlining the screening methodology. Huh7_Rluc_ cells were seeded in 384-well plates, infected with firefly luciferase-expressing *P. berghei* (*Pb*_Fluc_) sporozoites for 2 h, treated with 10 μM of each compound using an acoustic liquid handler, and incubated for a total of 2 days prior to performing the dual-luciferase reporter assay. The impact of each compound was assessed across 2–4 independent experiments (*N* = 2–4). (**B**) Robust Z′ (RZ′) values were calculated for each plate and for both firefly luciferase (Fluc, in green) and Renilla luciferase (Rluc, in blue) luminescence readouts. Plates with at least one RZ′ value below 0.2 (gray dashed line) were excluded from further analysis. (**C**) Fluc and Rluc luminescence values associated with each compound. Data are normalized and presented as mean values. 0% (black dashed line) and −100% correspond to no and complete inhibition, respectively. Green dashed lines indicate the Fluc (−84.24%) and Rluc (−41.64%) luminescence thresholds for the determination of hits, with publicly available compounds in the green shaded area selected for further characterization.

To identify new targets and chemical scaffolds with antiplasmodial activity, the dual-luciferase reporter assay was employed to screen a library of 6,044 compounds, partially overlapping with the previously published Novartis Moa Box collection (19). This library consists of bioactive compounds with annotated human targets, sourced from both public and internal data sets. Identifiers for retrieving chemical structures and associated information from public databases are provided for 4,273 of the 6,044 compounds (Data S1). Compounds were screened at a final concentration of 10 μM across 2–4 independent experiments (Fig. 2A and Data S1). As a quality control measure, robust Z′ (RZ′) values were calculated for each plate and luminescence signal (Fig. 2B and Data S1). The means and standard deviations of RZ′ for the Fluc and Rluc signals were 0.61 ± 0.21 and 0.76 ± 0.16, respectively, confirming the robustness and reliability of the assay (38–40). Plates with RZ′ values below 0.2 were excluded from further analysis. As expected from a bioactive collection (41–43), several compounds impacted liver-stage development and host cell viability (Fig. 2C). It is worth noting that a small subset of compounds induced an increase in the Rluc signal relative to DMSO-treated controls (i.e., 251 out of 6,044 compounds have Rluc values ≥ 100%) (Fig. 2C and Data S1), highlighting the need to confirm Rluc results using an orthogonal cytotoxicity assay. This effect likely reflects a known technical artifact and is consistent with a previous report showing that certain compounds, including staurosporine, can enhance CMV promoter activity (44), which drives Rluc expression in our system (Fig. S4). To select liver-stage inhibitors for follow-up studies, cutoffs were applied (see Materials and Methods), resulting in a set of 198 compounds with potent activity against *P. berghei* liver stages and minimal cytotoxicity (Fig. 2C). Out of these hits, 126 compounds were publicly available and are further characterized in this publication (Data S1). Collectively, these results suggest that screening the bioactive library enabled the identification of *Plasmodium* liver-stage inhibitors.

### Dose-response validation of hit compounds against *P. berghei* liver stages

**Fig 3:**
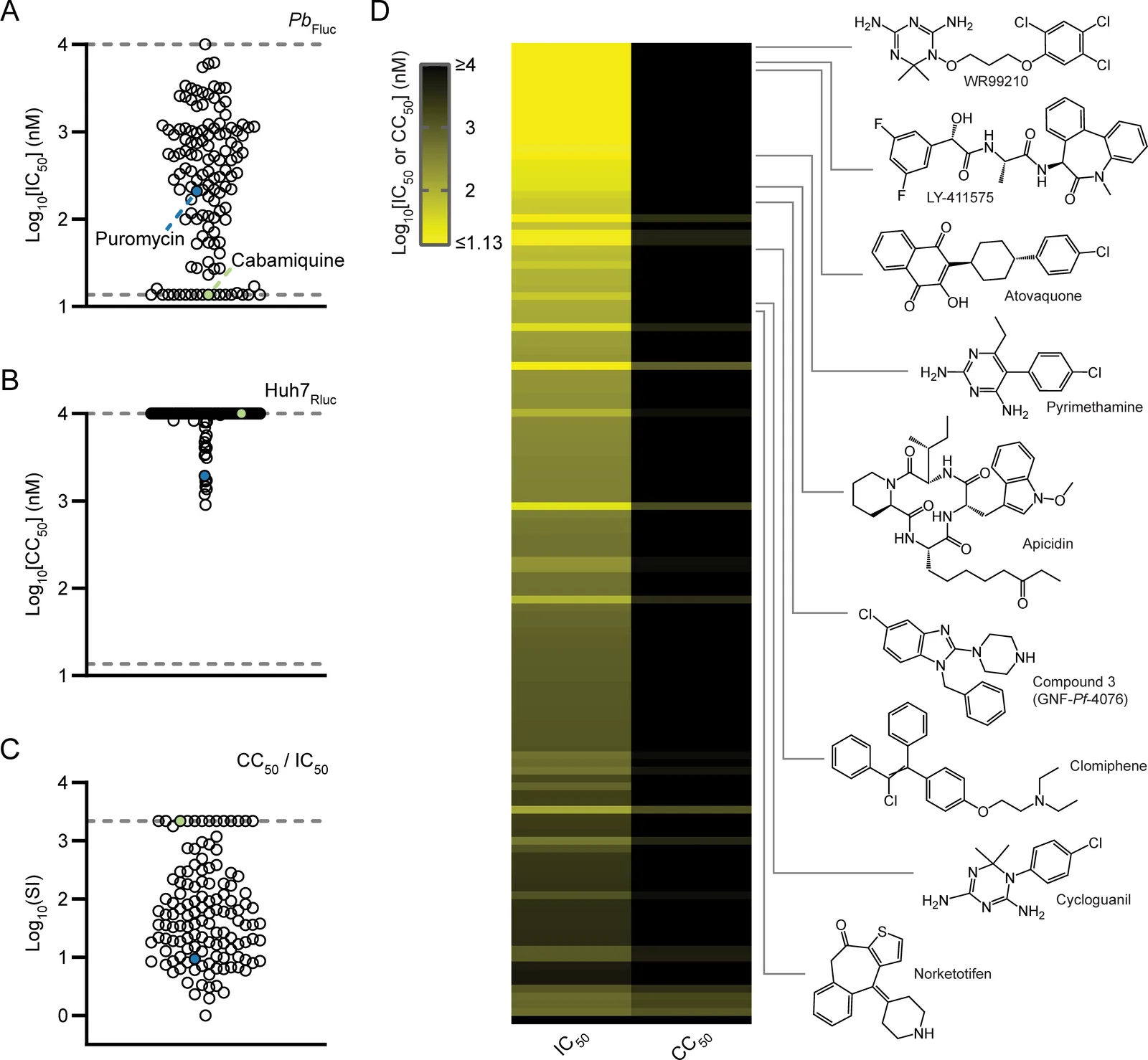
Dose-response validation of hit compounds against *P. berghei* liver stages. The dose-response activity of publicly available hit compounds was assessed against *P. berghei* liver stages at 2 days post-infection using the dual-luciferase reporter assay. Median IC_50_s (**A**), median CC_50_s (**B**), and approximate SIs (**C**) were determined from 2 to 4 independent experiments (*N* = 2–4). Cabamiquine (in green) and puromycin (in blue) served as antiplasmodial and cytotoxic controls, respectively. Limits of detection are illustrated by gray dashed lines. Values below or above the limit of detection were imputed to allow the determination of medians and SIs. (**D**) Heat map illustrating median IC_50_s and CC_50_s for hit compounds. Compounds are organized from top to bottom based on their SI. Molecular structures of compounds with known antiplasmodial activity are showcased. Some data are shared between this figure and Fig. 4 and 5.

The publicly available compounds identified in the single-point screen were validated through dose-response experiments using the dual-luciferase reporter assay (Fig. 3 and Data S1). These experiments enabled the determination of *Pb*_Fluc_ IC_50_s (Fig. 3A), Huh7_Rluc_ CC_50_s (Fig. 3B), and SIs (Fig. 3C), calculated as the ratio of CC_50_ to IC_50_, which reflects the window between cytotoxicity and antiplasmodial activity. As a benchmark, the cytotoxic control compound puromycin yielded an SI of 9.4, which was used to set an approximate SI cutoff of 10 for hit prioritization. Compounds were ranked by SI and visualized in a heat map, highlighting those with potent activity against *P. berghei* liver stages (Fig. 3D). A total of 99 compounds exceeded an SI of 10 (Data S1), indicating selective antiplasmodial activity. To validate cytotoxicity assessment, profiles of most compounds (122 out of 126) were evaluated using the orthogonal CTG assay (Fig. S5 and Data S1). CC_50 Rluc_ and CC_50 CTG_ values were largely consistent, with 83.61% and 94.26% of measurements falling within threefold and ninefold differences, respectively (corresponding to one and two steps in the serial dilution series). As an additional control, 20 compounds associated with single-point Rluc values < −80% at 10 µM were also tested, and all showed detectable cytotoxicity in the CTG assay (Fig. S5 and Data S1).

**Fig 4:**
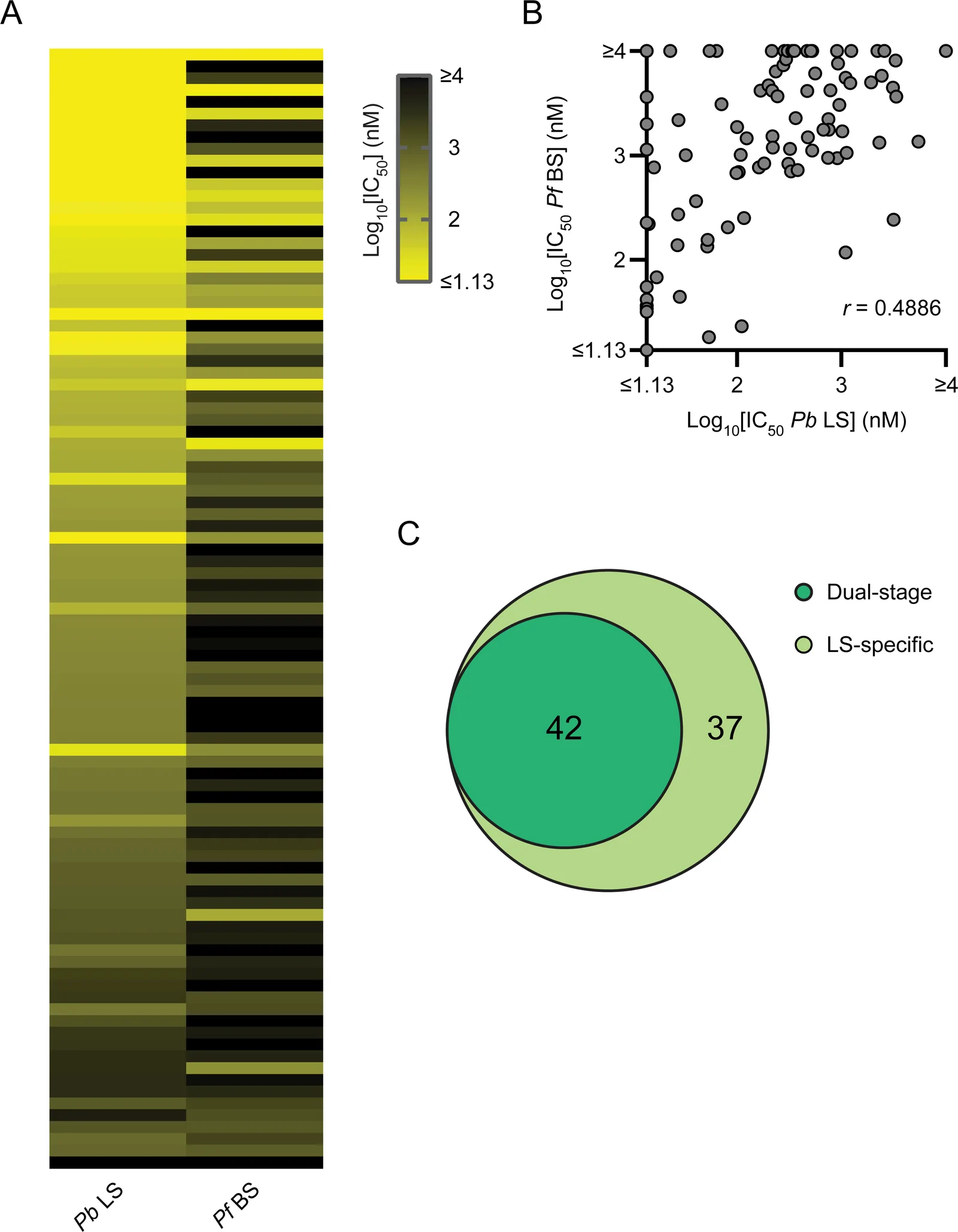
Characterization of hit compounds against *P. falciparum* asexual blood stages. (**A**) Heat map displaying median IC_50_s of hit compounds against *P. berghei* liver stages and *P. falciparum* blood stages. Median IC_50_s against *P. falciparum* blood stages were obtained from at least one experiment with technical duplicates. Values outside the limits of detection were imputed to calculate medians and harmonized between assays for graphical representation. (**B**) Graph showing the correlation between *P. berghei* liver-stage and *P. falciparum* blood-stage IC_50_s. The Pearson correlation coefficient (*R*) is shown. (**C**) Venn diagram showing numbers of compounds with dual-stage (IC_50_*Pf* BS / IC_50_*Pb* LS < 10) and liver stage-specific (IC_50_*Pf* BS / IC_50_*Pb* LS > 10) antiplasmodial activities. Only compounds with an SI (CC_50_ Huh7_Rluc_ / IC_50_*Pb* LS) > 10 were considered in panel C. Some data are shared between this figure and Fig. 3 and 5.

**Fig 5:**
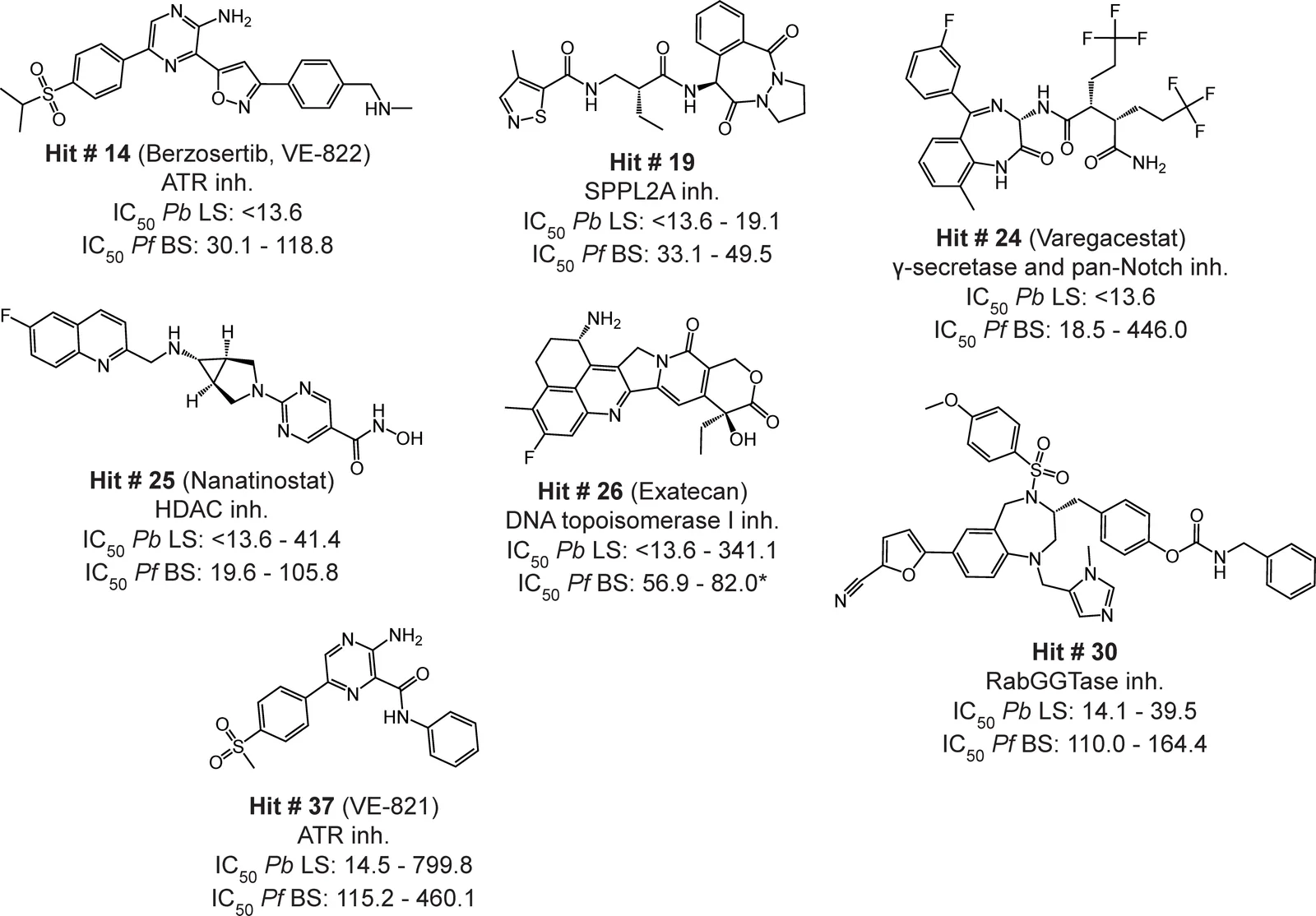
Dual-stage inhibitors of *Plasmodium* parasites. Structures of select dual-stage inhibitors are featured. Primary known mechanism of action in humans and ranges of IC_50_s (i.e., min − max nM values) against *P. berghei* liver stages and *P. falciparum* asexual blood stages are shown. Compounds were selected based on their antiplasmodial activity and the absence of detectable Rluc inhibitory activity in the dual-luciferase reporter assay (CC_50_ > 10 µM). IC50 values are from at least three independent experiments. An asterisk (*) denotes that one outlier was excluded from the corresponding IC_50_ range based on Grubbs’ test (α = 0.05) performed in GraphPad Prism. ATR, ataxia telangiectasia and Rad3-related; HDAC, histone deacetylase; RabGGTase, Rab geranylgeranyl-transferase; SPPL2A, signal peptide peptidase-like 2A. See Data S1 for single and averaged values from each independent experiment. Some data are shared between this figure and Fig. 3 and 4.

For many hit compounds, annotated mechanisms of action in human cells are publicly available (Data S1), which may facilitate downstream target identification (4, 7, 45, 46). While most hits were not previously known to exhibit antiplasmodial activity and may act through novel mechanisms, others are known inhibitors of *Plasmodium* liver stages (Fig. 3D) including LY-411575 (Hit # 7) (47, 48), pyrimethamine (Hit # 27) (11, 27, 28, 46), clomiphene (Hit # 47) (46), cycloguanil (Hit # 58) (28), and norketotifen (Hit # 59) (49). Notably, WR99210 (Hit # 5) (50, 51), LY-411575 (47), atovaquone (Hit # 9) (52, 53), pyrimethamine (7, 50), apicidin (Hit #32) (54), compound 3/GNF-*Pf*-4076 (Hit #40) (30, 55), cycloguanil (7), and norketotifen (49) have documented activity against asexual blood stages, suggesting that some of the newly identified compounds may possess multistage activity. In contrast, clomiphene has been reported to have liver stage-specific activity (46). Overall, these findings demonstrate that screening the bioactive library identified both known and potentially novel *Plasmodium* inhibitors. Together with the results presented in the previous sections, they further support the utility of the dual-luciferase assay for compound discovery.

### Profiling hit compounds against *P. falciparum* asexual blood stages

Given that several identified hits have known activity against *Plasmodium* asexual blood stages (Fig. 3D), an expanded set of compounds was tested to assess cross-stage efficacy (Fig. 4 and Data S1). Notably, a subset of *P. berghei* liver-stage inhibitors also demonstrated activity against *P. falciparum* blood stages (Fig. 4A), resulting in a moderate positive correlation between liver-stage and blood-stage IC_50_s (Pearson, *r* = 0.4886) (Fig. 4B). Among compounds with an SI > 10, we identified 42 dual-stage (IC_50_*Pf* BS / IC_50_*Pb* LS < 10) and 37 potential liver stage-specific (IC_50_*Pf* BS / IC_50_*Pb* LS > 10) inhibitors (Fig. 4C and Data S1). These findings suggest that a substantial fraction of the inhibitors target biological pathways essential for both liver-stage and asexual blood-stage development.

Conversely, compounds that exclusively inhibit *P. berghei* liver stages may act on host or parasite pathways uniquely required during liver-stage infection. Alternatively, they may exhibit species-specific activity across different *Plasmodium* parasites or constitute technical artifacts (56). Because the liver stage-specific inhibitors identified in this study are supported solely by data from the dual-luciferase reporter assay, further validation is required to confirm their biological relevance. Nevertheless, given the complementarity between the *P. berghei* liver-stage and the *P. falciparum* blood-stage assays, this study confidently identified a collection of dual-stage inhibitors of malaria.

### Dual-stage inhibitors of *Plasmodium* parasites

The chemical structures of the *Plasmodium* inhibitors highlighted in this study, together with annotations for several human targets, are publicly available (Data S1). Among the dual-stage inhibitors, notable examples include ATR inhibitors VE-822 and VE-821 (57, 58), a signal peptide peptidase-like 2A (SPPL2A) inhibitor (59), the γ-secretase and pan-Notch inhibitor Varegacestat (60), the histone deacetylase (HDAC) inhibitor Nanatinostat (61), the DNA topoisomerase I inhibitor Exatecan (62, 63), and a RabGGTase inhibitor (64, 65) (Fig. 5 and Data S1). While these compounds are known to act on host targets and pathways, their antiplasmodial activity may arise from the inhibition of *Plasmodium* homologs, particularly for validated parasite targets such as HDACs (54, 66) and DNA topoisomerases (67, 68). Moreover, knowledge of a compound’s human target can facilitate the identification of structurally related parasite proteins that may also be susceptible to inhibition. For example, we found that *P. falciparum* PI(4)K (PF3D7_0509800) shares structural features with human ATR (Fig. S6). Consistent with this, ATR inhibitors VE-822 and VE-821 (Fig. 5) exhibit structural similarity to the known *Plasmodium* PI(4)K inhibitor MMV390048 (69) and potently inhibit recombinant *P. vivax* PI(4)K *in vitro* (Fig. S6 and Data S1). These findings suggest that our dual-stage inhibitors may exert antiplasmodial effects through parasite targets. Overall, this study has identified a diverse set of dual-stage antiplasmodial compounds, offering valuable starting points for target identification and therapeutic development.

## DISCUSSION

The liver phase of *Plasmodium* infection represents a critical window for pharmacological intervention, complementing approaches targeting the symptomatic blood phase of infection (3, 4). During the liver phase, parasites undergo intracellular replication within hepatocytes, exploiting both host and parasite processes (5) that may be amenable to pharmacological modulation. However, effective liver-stage pharmacological interventions must inhibit parasite development without significant adverse effects on host cells and tissues. To expedite the discovery of non-cytotoxic antiplasmodials, we tailored a dual-luciferase reporter assay capable of simultaneously quantifying *Plasmodium* liver-stage development and host cell viability, enabling immediate exclusion of cytotoxic compounds (Fig. 1). Using this assay, we screened a chemogenetic library of small molecules with annotated human targets (Fig. 2 and Data S1) and identified novel inhibitors of liver-stage parasites (Fig. 3). Several of these compounds also suppressed *P. falciparum* asexual blood stages (Fig. 4 and 5), suggesting dual-stage activity.

Although chemogenetic libraries provide a powerful approach for accelerating target identification (19, 41), dissecting compound mechanisms during liver-stage infection remains challenging. Homologous proteins in host and parasite can complicate target attribution and validation, particularly when phenotypes may arise from effects on either or both. However, our compounds that inhibit both liver and blood stages are hypothesized to act on parasite targets conserved across developmental stages and species, rather than on host pathways, as red blood cells lack nuclei and most organelles and therefore offer fewer host targets (70). For instance, Hit # 30 may inhibit *Plasmodium* RabGGTase (Fig. 5), potentially disrupting Rab protein prenylation and membrane localization (71, 72), suggesting that prenyltransferases may represent a broader class of antiplasmodial targets (65, 73). As another example, human ATR inhibitors VE-822 and VE-821 may act on parasites by inhibiting *Plasmodium* PI(4)K (Fig. S6). These examples illustrate how annotated human targets, especially when integrated with structure-based approaches, can inform parasite target discovery and accelerate mechanistic insight.

In addition to dual-stage inhibitors, we also identified compounds with potential liver stage-specific activity. These may act on parasite pathways uniquely required for liver-stage development, on host pathways, or reflect species-specific biology, as liver-stage activity was assessed in *P. berghei* and blood-stage activity in *P. falciparum*. An example of a host-directed mechanism is the inhibition of liver stages by the cholesterol trafficking and biosynthesis inhibitor U18666A (74, 75) (Hit # 12, Data S1), consistent with the established role of host cholesterol in supporting parasite development (76, 77). Our results also suggest that liver-stage parasites may be sensitive to inhibition of host hypoxia-inducible factor (HIF)-1α (Moracin P, Hit #22; Data S1), a transcription factor mediating cellular responses to hypoxia in addition to its roles under normoxic conditions (78–80). Consistent with this finding, a previous study indicated that hypoxia promotes the development of *Plasmodium* liver stages through mechanisms partly mediated by HIF-1α (81). Interestingly, HIFs and the hepatic oxygen gradient have been implicated in the metabolic zonation of the liver (82), with the perivenous lobule zone—characterized by lower oxygen tension—being more permissive to liver-stage development (81, 83). As such, HIF-1α may contribute to the differential susceptibility of hepatocyte subpopulations to *Plasmodium* infection. Overall, these findings highlight potential host-parasite interactions that warrant further investigation.

The chemically diverse hits identified in this study broaden the antiplasmodial discovery space and include potent, low-toxicity inhibitors with structures and annotations that support mechanism-of-action deconvolution and medicinal chemistry optimization. However, it should be noted that the results of this study should be interpreted in the context of variations in the number of independent experiments performed for each hit compound and assay. Although further profiling is needed to assess their suitability, some of these hits may serve as starting points for developing chemotypes with potential relevance to MMV’s long-acting chemoprevention strategy (84). In summary, this study lays the groundwork for future efforts to validate novel targets, optimize lead compounds, and inform the design of next-generation antiplasmodial drugs for both prevention and treatment.

## SUPPLEMENTAL MATERIAL

Data S1 aac.01613-25-s0001.xlsx Data associated with this study.

Supplemental figures aac.01613-25-s0002.pdf Fig. S1 to S6.

ASM does not own the copyrights to Supplemental Material that may be linked to, or accessed through, an article. The authors have granted ASM a non-exclusive, world-wide license to publish the Supplemental Material files. Please contact the corresponding author directly for reuse.
