# Supplementary material for: Screening of a chemogenetic library unveils dual-stage antiplasmodials with low cytotoxicity
Source: Antimicrob Agents Chemother. 2026 Oct 5;70(11):e01613-25. doi: 10.1128/aac.01613-25 (PMC13731184; doi:10.1128/aac.01613-25)
Supplement: Supplementary file 1 [file aac.01613-25-s0002.pdf]

Screening of a chemogenetic library unveils dual-stage antiplasmodials with low cytotoxicity

SUPPLEMENTAL FIGURES

Laura Torres[a], Matthew E. Fishbaugher[b], Melanie Lam[a], Alexander T. Chao[b], Calla Martyn[c], Luying Pei[b], Debapriya Sengupta[b], Maxime Dauphinais[d], Jonathan E. Gable[b], Srinivasa P. S. Rao[b], Steffen Renner[e], W. Armand Guiguemde[b], Sebastian A. Mikolajczak[b], Anke Harupa[b#], Gabriel Mitchell[a#]

[a] Open Innovation at Global Health Disease Area, Biomedical Research, Novartis, Emeryville, CA, USA

[b] Global Health Disease Area, Biomedical Research, Novartis, Emeryville, CA, USA

[c] Discovery Sciences, Biomedical Research, Novartis, Emeryville, CA, USA

[d] Global Discovery Chemistry, Biomedical Research, Novartis, Emeryville, CA, USA

[e] Discovery Sciences, Biomedical Research, Novartis, Basel, Switzerland

[#]Corresponding authors: anke.harupa-chung@novartis.com and gabrielmitchell2@gmail.com

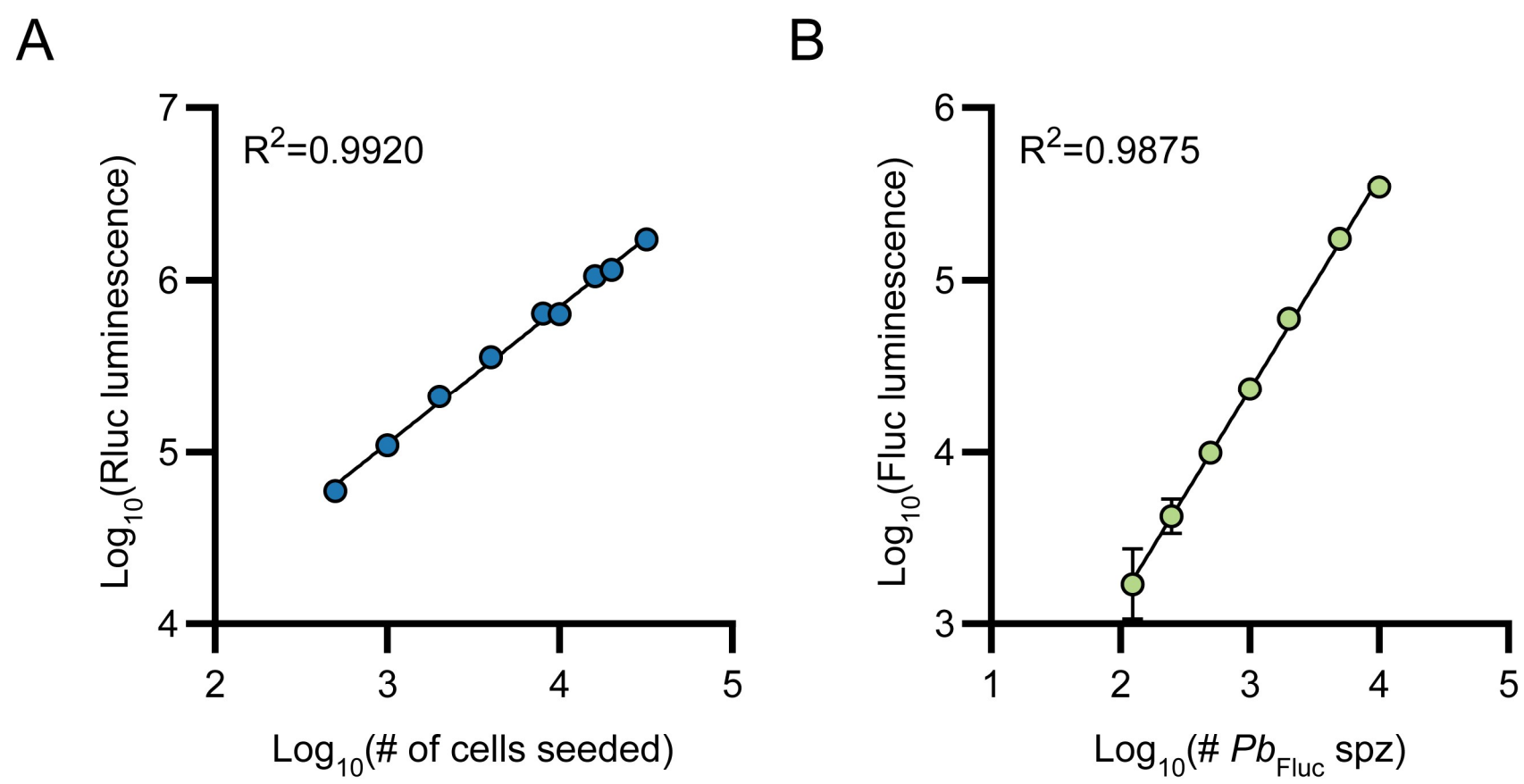

Figure S1. (A) Renilla luminescence (Rluc) as of function of the number of Huh7$_{\text{Rluc}}$ cells seeded per well of a 384-well plate. The number of cells seeded ranged from 500 to 32,000. Results were obtained from uninfected cells, using the Rluc assay, and are represented as means and SDs from one experiment (N=1) performed with six technical replicates. (B) Firefly luciferase (Fluc) luminescence as of function of the number of Fluc-expressing *P. berghei* (*Pb*$_{\text{Fluc}}$) sporozoites used to infect cells for 2 days. The number of sporozoites ranged from 125 to 10,000. Results were obtained using the dual-luciferase reporter assay and are represented as means and SDs from one experiment (N=1) performed with four technical replicates. Coefficients of determination (R^2) from simple linear regressions are shown.

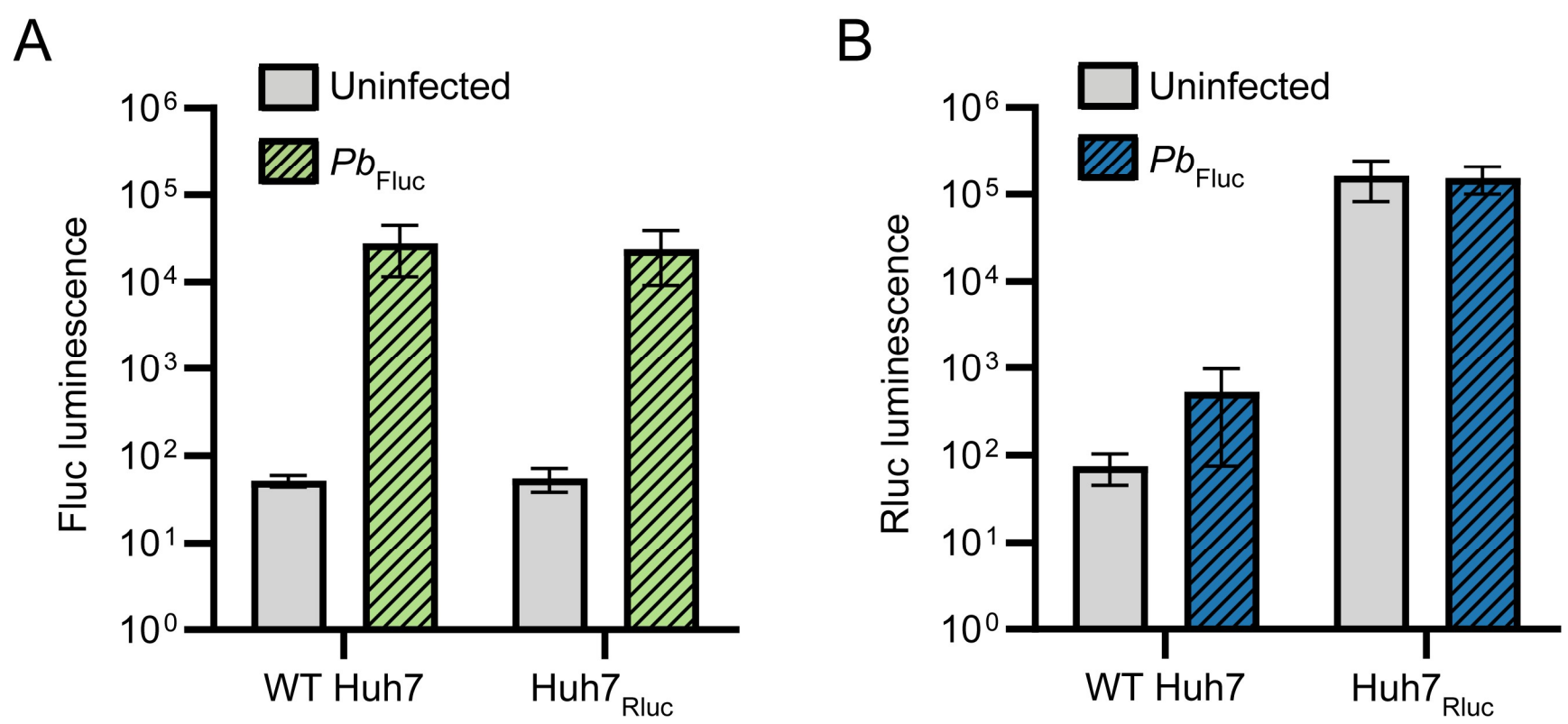

Figure S2. Fluc (A) and Rluc (B) luminescence of WT Huh7 and $Huh7_{Rluc}$ cells infected with Pb_{Fluc} for 2 days. Cells were seeded at a density of 10,000 cells per well one day prior to infection with 2,000 sporozoites. Signal-to-noise ratios were 392 ± 150-fold for Fluc and 775 ± 563-fold for Rluc. Results are unnormalized and presented as means and SEMs from two independent experiments (N=2, with 5 technical replicates).

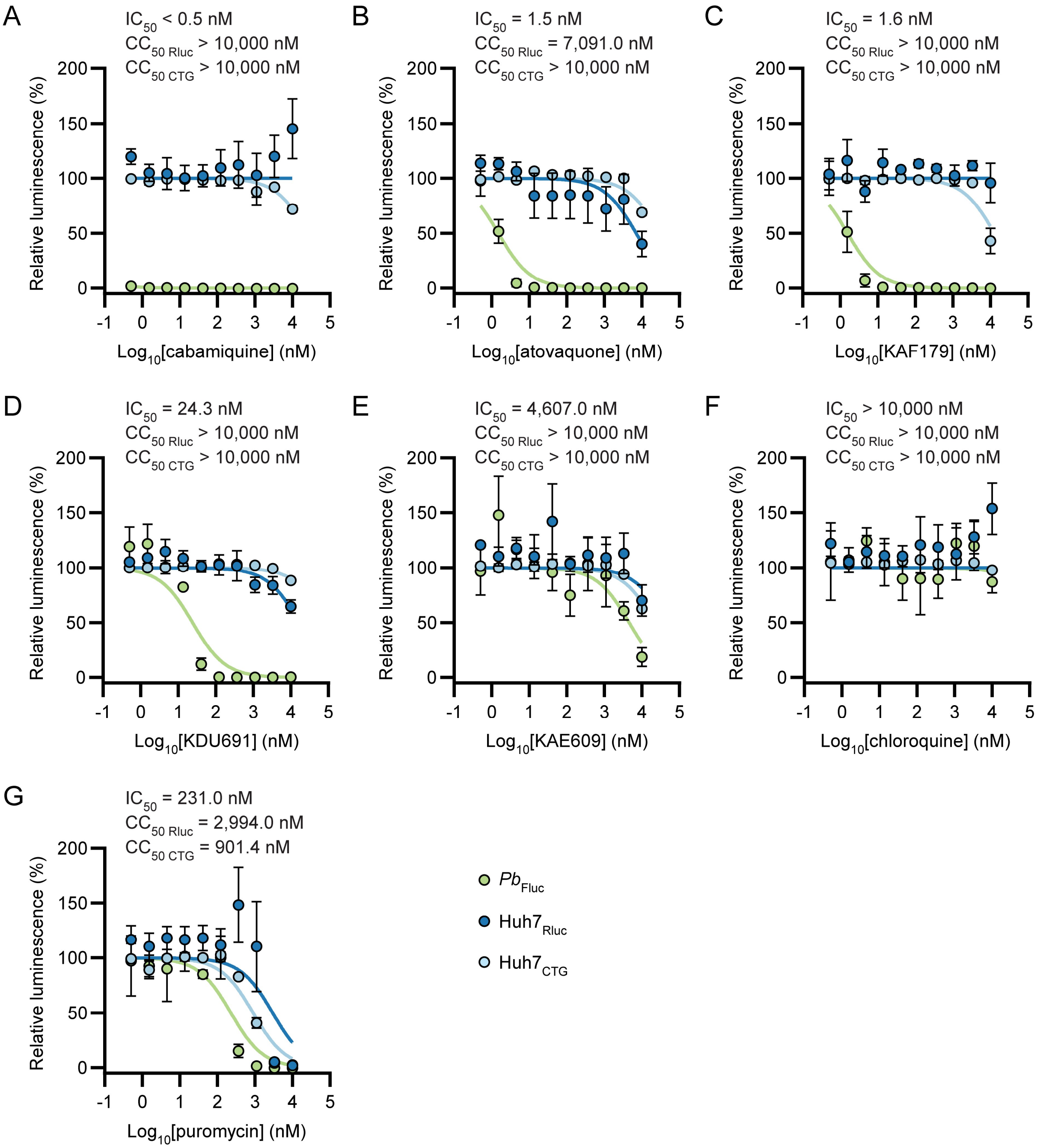

Figure S3. Dose-response graphs showing the impact of cabamiquine (A), atovaquone (B), KAF179 (C), KDU691 (D), KAE609 (E), chloroquine (F) and puromycin (G) on *P. berghei* (Pb_{Fluc}) liver-stage development and Huh7 ($\text{Huh7}_{\text{Rluc}}$) viability as determined using the dual-luciferase reporter assay 2 days post-infection. Cytotoxicity of compounds was also assessed in uninfected cells using the CellTiter-Glo® (CTG) assay. Results are presented as means and SEMs from three to four independent experiments (N =3-4) performed with two to four technical replicates. IC_{50} (Pb_{Fluc}) and CC_{50} ($\text{Huh7}_{\text{Rluc}}$ and Huh7_{CTG}) values were determined from curve fits performed on pooled data across independent experiments. See Supplemental Data S1 for single

and averaged IC50 and CC50 values from each independent experiments. 100% corresponds to DMSO-treated samples. Data were analyzed using GraphPad Prism. Some data are shared between Fig. 1 and Fig. S3.

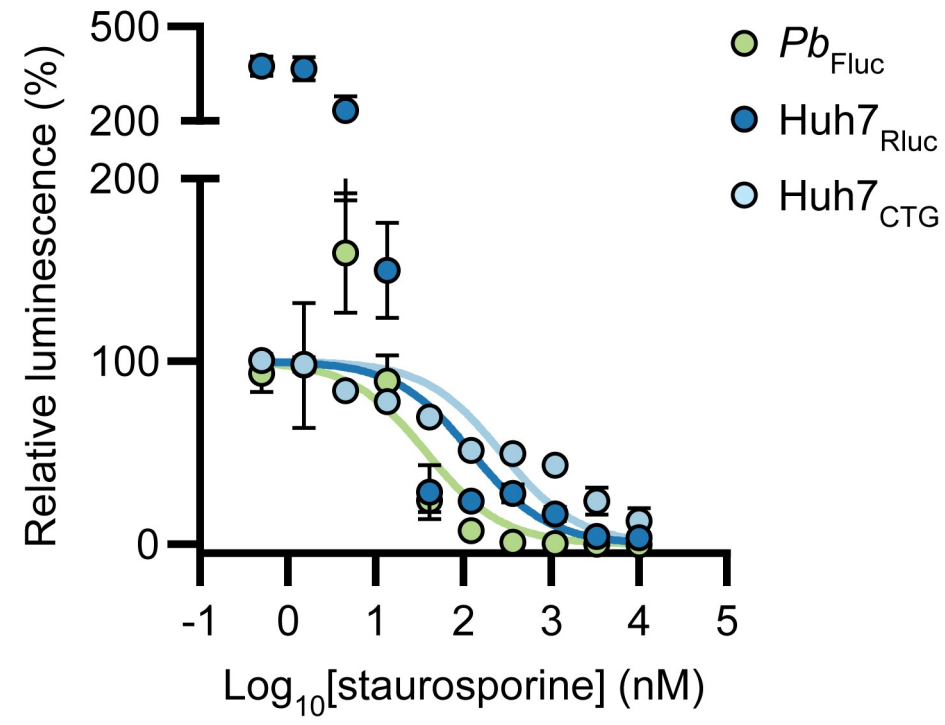

Figure S4. Dose-response graph showing the effect of staurosporine on *P. berghei* (*Pb*$_{\text{Fluc}}$) liver-stage development and Huh7 (Huh7$_{\text{Rluc}}$) viability as determined using the dual-luciferase reporter assay 2 days post-infection. Cytotoxicity was also evaluated in uninfected cells using the CellTiter-Glo® (CTG) assay. Results are presented as means and SEMs from three to four independent experiments (N=3-4) performed with technical duplicates. 100% corresponds to DMSO-treated samples. Data were analyzed using GraphPad Prism.

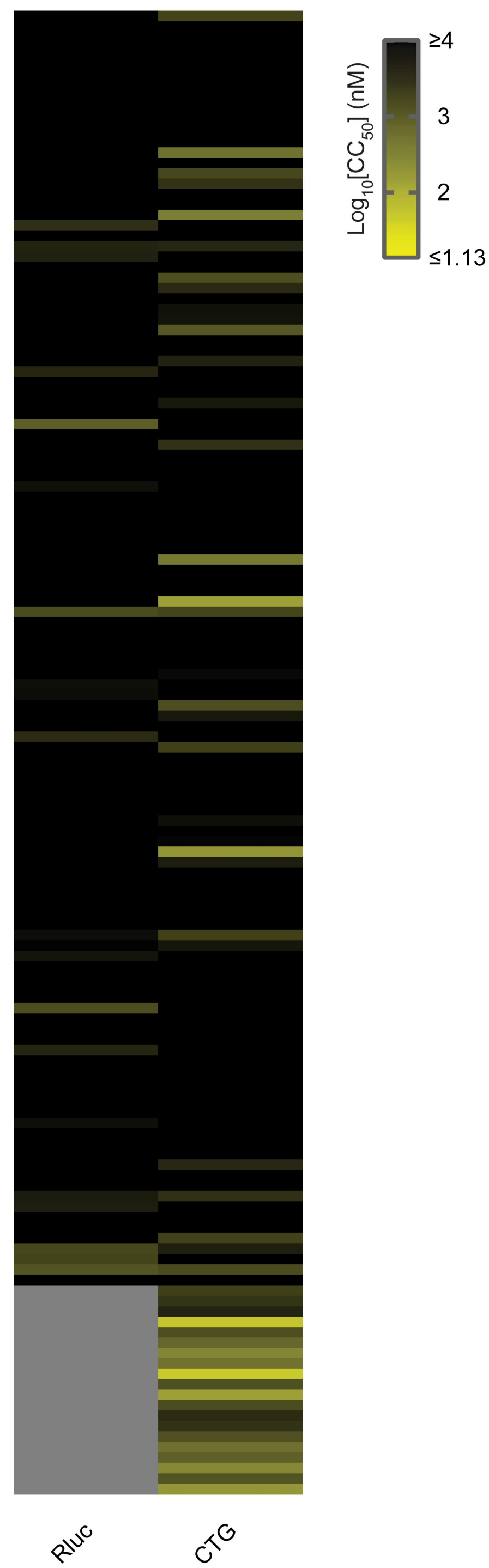

Figure S5. Heat map comparing the median CC_{50} values of selected hit compounds in $Huh7_{Rluc}$ cells as determined using the dual-luciferase reporter assay (Rluc column) and CellTiter-Glo (CTG column). $CC_{50\ CTG}$ values for compounds with single-point Rluc values < -80%, therefore presumed cytotoxic at 10 µM, were also included. The $CC_{50\ Rluc}$ values were not determined for compounds with single-point Rluc values < -80%, as indicated in gray on the heat map. CTG results are from three to four independent experiments. Some data are shared with Fig. 3.

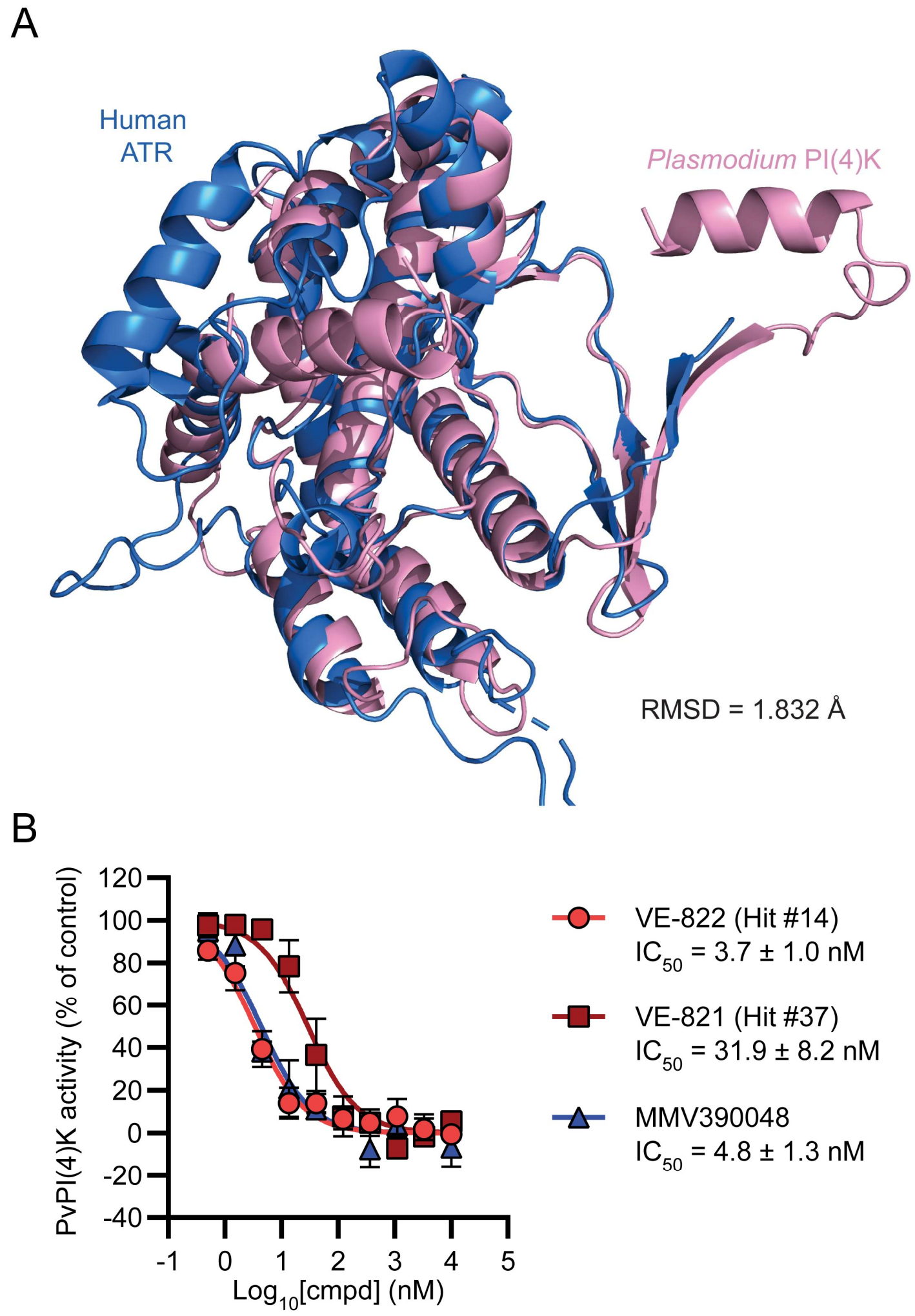

Figure S6. (A) Structural alignment of *P. falciparum* PI(4)K (amino acid 1280-1556, in pink) and human ATR (amino acid 2320-2632, in blue). The alignment was performed using PyMol as described in the Materials and Methods. RMSD, root mean square deviation. (B) Effect of ATR inhibitors VE-822 (Hit #14) and VE-821 (Hit #37) and a control *Plasmodium* PI(4)K inhibitor (MMV390048) on the activity of recombinant *P. vivax* (Pv) PI(4)K. Results are presented as means and SEMs from three independent experiments (N=3) performed with technical duplicates. 100% corresponds to DMSO-treated samples. Curve fitting and IC_{50} determination were performed in GraphPad Prism. See Supplemental Data S1 for values from each independent experiments.
